# Rapid Screening of Methicillin-Resistant *Staphylococcus aureus* Using MALDI-TOF MS and Machine Learning: A Randomized, Multicenter Study

**DOI:** 10.1021/acs.analchem.5c01286

**Published:** 2025-07-16

**Authors:** Dongeun Yong, Jeong Su Park, Kyungnam Kim, Donggun Seo, Dong-Chan Kim, Jae-Seok Kim, Jong-Min Park

**Affiliations:** † Department of Laboratory Medicine and Research Institute of Bacterial Resistance, 37991 Yonsei University College of Medicine, Seoul 03722, Republic of Korea; ‡ Department of Laboratory Medicine, Seoul National University Bundang Hospital, Seoul National University College of Medicine, Gyeonggi-do 13620, Republic of Korea; § NQ-LAB Co., ltd., Gyeonggi-do 16827, Republic of Korea; ∥ Department of Laboratory Medicine, Kangdong Sacred Heart Hospital, Hallym University College of Medicine, Seoul 05355, Republic of Korea; ⊥ Major in Materials Science and Engineering, School of Future Convergence, 26727 Hallym University, Gangwon-do 24252, Republic of Korea; # Interdisciplinary Program of Nano-Medical Device Engineering and Integrative Materials Research Institute, 26727 Hallym University, Gangwon-do 24252, Republic of Korea

## Abstract

Methicillin-resistant *Staphylococcus aureus* (MRSA) is a major cause of healthcare-associated infections including bacteremia. The rapid detection of MRSA is essential for prompt treatment and improved outcomes. However, traditional MRSA screening and confirmatory tests based on bacterial cultures with antimicrobial susceptibility tests and/or molecular diagnostics are time-consuming (>2 days), labor-intensive, and costly. We report that AMRQuest software, which was developed using logistic regression-based machine learning and matrix-assisted laser desorption/ionization-time-of-flight spectra of *S. aureus* isolates, can be successfully implemented in clinical microbiology laboratories to screen MRSA and identify bacterial species simultaneously, with the cefoxitin disk diffusion test as a reference. Analytical sensitivity, specificity, percent agreement, and Cohen’s kappa values were calculated to determine the accuracy of the AMRQuest software. The minimum sample size of the testing set for statistical analysis was determined considering the local prevalence of MRSA infections. MRSA screening was performed using 537 consecutive *S. aureus* isolates, including 231 MRSA and 306 methicillin-susceptible *S. aureus* isolates, from three tertiary-care hospitals. The results from the AMRQuest software were similar to those obtained using the reference method, cefoxitin disk diffusion testing, making it a powerful method for the rapid detection of MRSA prior to traditional antibiotic resistance testing.

## Graphical abstract

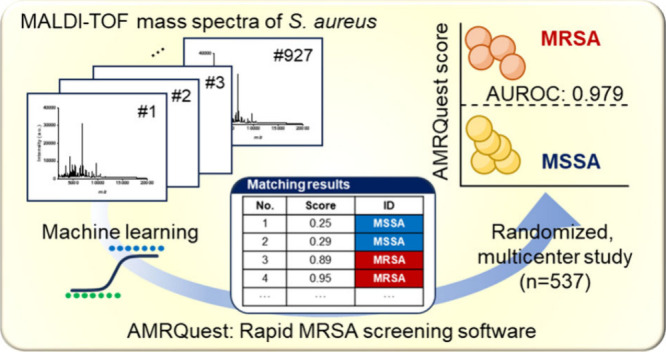

Methicillin-resistant *Staphylococcus aureus* (MRSA) is a major cause of healthcare-associated infections.^1^ Infectious diseases caused by MRSA tend to occur more frequently in patients who undergo invasive procedures or are immunocompromised. In hospitals, MRSA threatens the lives of patients by causing bacteremia, pneumonia, surgical wound infection, and skin diseases, even in healthy individuals.^2,3^ There is a risk of harm to the patient due to failure of the initial treatment when antibiotic-resistant bacteria are determined to be susceptible. Additionally, this can lead to unnecessary treatment costs and manpower usage because antibiotic-susceptible bacteria are resistant. Therefore, rapid detection of MRSA is essential for the prompt and appropriate treatment of MRSA infections to improve treatment outcomes.

Broth dilution and disk diffusion tests are widely used for MRSA screening and confirmatory testing.^4^ However, these tests require at least 2 days to obtain results. Molecular diagnostics involve same-day PCR, sequencing, and DNA chip detection of the staphylococcal cassette chromosome *mec* (SCC*mec*), a mobile genomic element containing the *mecA* gene that induces methicillin resistance in *S. aureus.* ^5^ However, these methods are costly and labor-intensive.

Matrix-assisted laser desorption/ionization time-of-flight mass spectrometry (MALDI-TOF MS) has recently been used in clinical microbiology laboratories to identify various pathogens based on protein and peptide profiles.^6–8^ Moreover, MALDI-TOF MS can accelerate the detection of resistance compared with conventional antibiotic susceptibility tests.^9–12^ A small peptide (PSM-*mec*), which is encoded by SCC*mec* types II, III, and VIII and is visible at *m*/*z* 2415, can be used for MRSA screening using MALDI-TOF MS.^13^ However, this method was only effective for coagulase-negative staphylococci because only 29.4% of MRSA isolates contained the PSM-*mec* peptide.

In this study, AMRQuest software was developed to screen for MRSA and identify bacterial species simultaneously. The AMRQuest software provides a score that represents the likelihood that the bacterial isolate is MRSA by comparing the MALDI-TOF spectra of *S. aureus* with the database using a machine learning technique. We embedded the AMRQuest software into the MALDI-TOF MS with a bacterial identification system and used it to identify *S. aureus* isolates from patients and perform methicillin-resistance testing simultaneously, enabling faster treatment of patients with severe MRSA infections and preventing unnecessary antimicrobial overuse.

## Experimental Section

### Development of AMRQuest Software

AMRQuest software was developed to screen for MRSA based on machine learning techniques and statistical criteria for MRSA classification (Figure 1). First, the AMRQuest software was trained using a machine learning technique for MRSA screening. For training, 430 MRSA and 497 methicillin-susceptible *S. aureus* (MSSA) isolates were identified using the oxacillin disk diffusion test and SCC*mec* typing by PCR. Mass spectra of each *S. aureus* isolate were obtained using MicroIDSys LT MALDI-TOF system (ASTA, Suwon, Korea), as described in Supporting Information. The mass spectra of each isolate were processed in the following order: quality control, smoothing, baseline correction, intensity calibration, peak detection, and calculation of the intensity matrix, using the MALDIquant package version 1.17 function of R version 3.4.3.^14^ Subsequently, the feature matrix, which was composed of the mass values and intensities of the universal peaks, was obtained by binning the spectra in the *m*/*z* range of 5. To evaluate the contribution of the features in screening for MRSA, SHAP using the shap package^15^ and ANOVA analyses for each binned mass range were performed for both the MRSA and MSSA groups. Finally, the AMRQuest software was configured to load the MALDI-TOF mass spectrum of the *S. aureus* isolate and identify MRSA using the AMRQuest score, which was determined as the likelihood of the isolate being MRSA using a machine learning algorithm.

**Figure 1:**
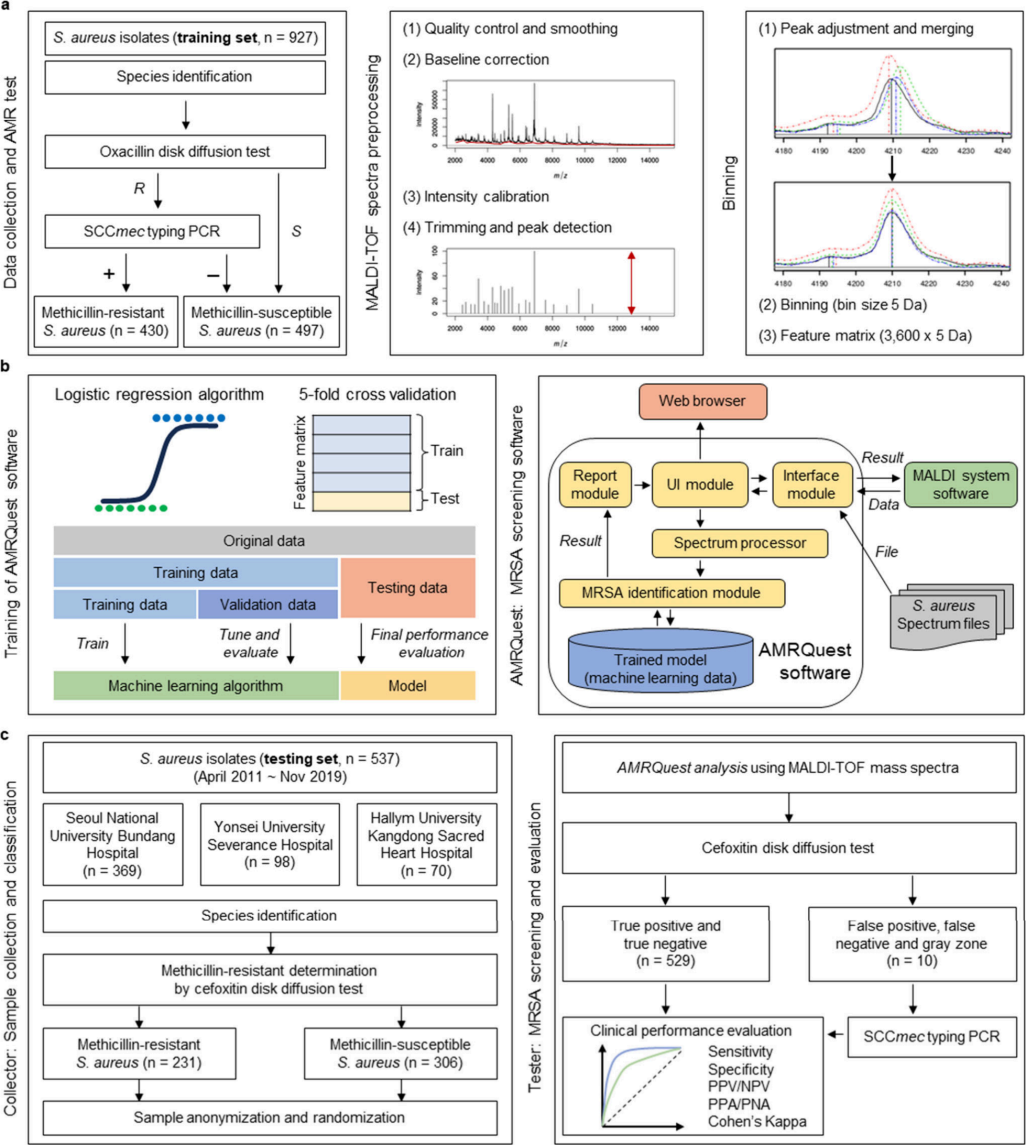
Workflow of the AMRQuest software which is the MALDI-TOF MS-based methicillin-resistant *S. aureus* (MRSA) screening system. (a) Processing of MALDI-TOF mass spectra for machine learning. (b) Training process and structure of AMRQuest software. AMRQuest software was designed as a module that can use MALDI-TOF spectra after *S. aureus* identification without additional MALDI process. (c) Randomized, single-blind study design for clinical evaluation of AMRQuest software. Minimum number of MRSA and methicillin-susceptible *S. aureus* (MSSA) isolates were calculated by using meta-analysis of the disease prevalence in Korea.

### Collection of Bacterial Isolates for Testing Set

This study was conducted between April 2020 and September 2020 at three university-affiliated teaching hospitals: Yonsei University Severance Hospital (Seoul, Republic of Korea), Hallym University Kangdong Sacred Heart Hospital (Seoul, Republic of Korea), and Seoul National University Bundang Hospital (Seongnam, Republic of Korea). In total, 537 *Staphylococcus aureus* strains were isolated from clinical blood cultures from April 2011 to November 2019 at three hospitals, as shown in the optimal sample size calculation described in the Supporting Information. All *S. aureus* isolates were identified by a MicroIDSys LT MALDI-TOF MS with MicroID CoreDB version 1.27.04 (ASTA, Suwon, Korea). To distinguish between MRSA and MSSA, antibiotic susceptibility of each *S. aureus* isolate was determined using the conventional methods including minimum inhibitory concentration (MIC) for oxacillin or cefoxitin, conducting the disk diffusion test with 30 μg of cefoxitin disk, or detecting the *mecA* gene. According to the CLSI guideline,^16^ the isolates were determined as MRSA if the MIC was greater than or equal to 4 and 8 μg mL^–1^ for oxacillin and cefoxitin, respectively, inhibition zone diameter around the cefoxitin disk (30 μg) was less than or equal to 21 mm, or the *mecA* gene were detected.

To conduct a randomized, single-blind study, *S. aureus* isolates were randomly labeled and delivered to a different “tester” hospital from the “collector” hospital that isolated the *S. aureus* to conduct MRSA screening using AMRQuest, as shown in Figure 1c.

### MRSA Screening Using AMRQuest Software

Prior to MRSA screening, the randomized *S. aureus* isolates were identified again using the MALDI-TOF MS and stored at–80 °C in the tester hospital. For MALDI-TOF bacterial identification, each isolate was grown on a blood agar (Shinyang Diagnostics, Siheung, Korea) for 12–18 h at 35 °C. Mass spectra of each *S. aureus* isolate were obtained and identified in the same way as for the training set.

MRSA screening using AMRQuest was performed at the Yonsei University Severance Hospital and Hallym University Kangdong Sacred Heart Hospital when the isolate was identified as *S. aureus*. Mass spectra of the identified *S. aureus* were exported to AMRQuest software and determined as either MRSA or MSSA. The AMRQuest score of each *S. aureus* isolate, which represents the likelihood of the sample being MRSA according to the machine learning algorithm, was used to distinguish between MRSA and MSSA isolates. *S. aureus* isolates with an AMRQuest score ≥ 0.5, were classified as MRSA, while isolates with a score <0.5, were classified as MSSA. To indicate uncertainty about scores around the cutoff score, the gray zone (i.e., low-confidence prediction range) was set to the range of cutoff score ± 0.1, i.e., 0.4–0.6.

### Evaluation of Clinical Performance of AMRQuest Software

To evaluate the clinical performance of AMRQuest, diagnostic performance parameters, including PPV, NPV, sensitivity, specificity, and Cohen’s kappa (*K*), were calculated from the results of AMRQuest and compared to those of conventional tests and the cefoxitin disk test, following the Evaluation Guideline of Clinical Performance for *In Vitro* Diagnostic Device from the Korean National Institute of Food and Drug Safety Evaluation.^17^ The clinical evaluation was conducted in two stages. First, the PPV and NPV of AMRQuest were calculated to determine its applicability in clinical microbiology laboratories to screen for MRSA. The targeted PPV was 84.8%, and the lower bound of the 95% confidence interval was greater than or equal to 70.3%. Similarly, the targeted NPV was 80.3% and the lower bound of the 95% confidential interval needed to be greater than or equal to 73.7%. Second, the PPA (sensitivity), PNA (specificity), and overall percent agreement were compared with the cefoxitin disk diffusion test results, which served as the standard for the AMRQuest software test results, and were performed by the tester hospital after sample randomization and anonymization. Then, the clinical performance of AMRQuest was evaluated using Cohen’s kappa (*K*) that was obtained by using the following equations and the parameters:

t=a+b+c+d 1

Pr(a)=(a+d)/t 2

$$\mathrm{Pr}\left(e\right)=\left(a+c\right)\left(b+d\right)/t^{2}+\left(a+b\right)\left(c+d\right)/t^{2}$$ 3

K=[Pr(a)−Pr(e)]/[1−Pr(e)] 4

Here, *a*, *b*, *c*, and *d* represent the true-positive, false-positive, false-negative, and true-negative values, respectively.

### SCC*mec* Typing PCR

Isolates that were identified as false negatives or false positives were inspected using SCC*mec*A typing PCR, as described previously.^18^ Briefly, DNA was isolated from *S. aureus* colonies using a HiYield Genomic DNA Mini Kit (Real Biotech Corporation, Banqiao City, Taiwan), according to the manufacturer’s instructions. PCR was performed using QIAGEN Multiplex PCR Master Mix (QIAGEN, Hilden, Germany) and SCC*mec* element-type primers.^19^

### Statistical Analysis

All experiments were randomized and single-blinded using the same setup. Potential biomarkers were identified by ANOVA and the SHAP method using the Shap package, which ranked the significance of the feature *m*/*z* ranges.^4,15^ MedCalc software version 20.019 for Windows (MedCalc Software Ltd.) and OriginPro 2022 software version 9.9.0.225 for Windows (OriginLab Corp.) were used for all the statistical analyses. The Mann–Whitney unpaired test was used to examine the differences in scores between MRSA and MSSA. AUC and PR curves were used to evaluate the diagnostic ability of the AMRQuest score as a criterion for MRSA screening.

### Ethical Approval

The research protocol was approved by the Institutional Review Board (IRB) of Severance Hospital (IRB number: 4-2019-1195). All study procedures were performed in accordance with the relevant guidelines and regulations. All clinical samples were anonymized before the cefoxitin disk diffusion test and clinical evaluation using the AMRQuest software for a single-blinded study.

## Results and Discussion

### Development and Training of AMRQuest Software

*S. aureus* isolates for the development and training of a machine learning-based MRSA screening software, AMRQuest, were collected as a training set containing 927 *S. aureus* isolates, independent of the testing set. The feature matrix from the MALDI-TOF mass spectra of the training set–430 isolates of MRSA and 497 isolates of MSSA was used to train the machine-learning-based MRSA screening software AMRQuest. MALDI-TOF MS was performed in the mass range of 2,000–20 000 Da to identify *S. aureus*. After identification, each spectrum was prepared as a feature matrix for training via peak adjustment, merging, and binning with an *m*/*z* range of 5 (Figure 1a). The binned spectrum samples were randomly split into training and testing data sets in a ratio of 4:1 to train the logistic regression machine learning algorithm. The AMRQuest software, as an independently operating clinical software tool, was configured to load the MALDI-TOF mass spectra of the *S. aureus* isolate, as shown in Figure 1b. For the testing set, 537 *S. aureus* isolates were collected from three tertiary care hospitals according to the calculation of the minimum sample size based on a meta-analysis^20^ and disease prevalence in Korea^21^ as described in Supporting Information. In total, 98, 70, and 369 *S. aureus* isolates were collected at Yonsei University Severance Hospital, Hallym University Kangdong Sacred Heart Hospital, and Seoul National University Bundang Hospital, respectively. A total of 231 MRSA isolates were identified using the cefoxitin disk diffusion test, according to the CLSI guidelines.^16^ All MRSA and MSSA isolates were numbered randomly and delivered from the “collector” hospital to the “tester” hospital for MRSA screening and clinical performance evaluation by AMRQuest software, as shown in Figure 1c.

Twenty features of *m*/*z* range with the highest contribution to distinguishing between MRSA and MSSA from logistic regression were extracted using the Shapley additive explanation (SHAP) method and ANOVA, as shown in Figure 2. The SHAP value, based on the Shapley value in game theory, indicates the contribution of each feature in the machine learning model to the prediction.^15^ The SHAP value for each spot indicated the contribution of the feature value or average peak intensity of that mass range to the determination of MRSA using AMRQuest. As shown in Figure 2a, the *m*/*z* ranges of 3005–3010, 3010–3015, and 4810–4815, which indicated the highest average absolute SHAP values, were effectively used for MRSA screening by logistic regression. Most of the feature *m*/*z* values with the highest contributions were distributed below 10 000 Da. Analysis of variance (ANOVA) was performed to analyze the upregulation of the average intensity in the individual feature mass range. As shown in Figure 2b, the average intensities in the *m*/*z* ranges 2410–2425, 2430–2445, 2450–2460, 3890–3900, 4045–4050, 4810–4820, and 5240–5245 were upregulated in MRSA, whereas the average intensity in the *m*/*z* range 5500–5530 was upregulated in MSSA. Among the features determined to be upregulated in MRSA and MSSA by ANOVA, *m*/*z* 2410–2420, 3890–3900, 4045–4050, 4810–4820, and 5510–5525 showed similar contributions in determining MRSA and MSSA in the SHAP plot. These results suggest that individual features with significant upregulation in MRSA or MSSA can be used as effective features in logistic regression-based MRSA screening. However, only three features were found to contribute significantly to both individual marker (ANOVA) and multimarker (SHAP) screening.

**Figure 2:**
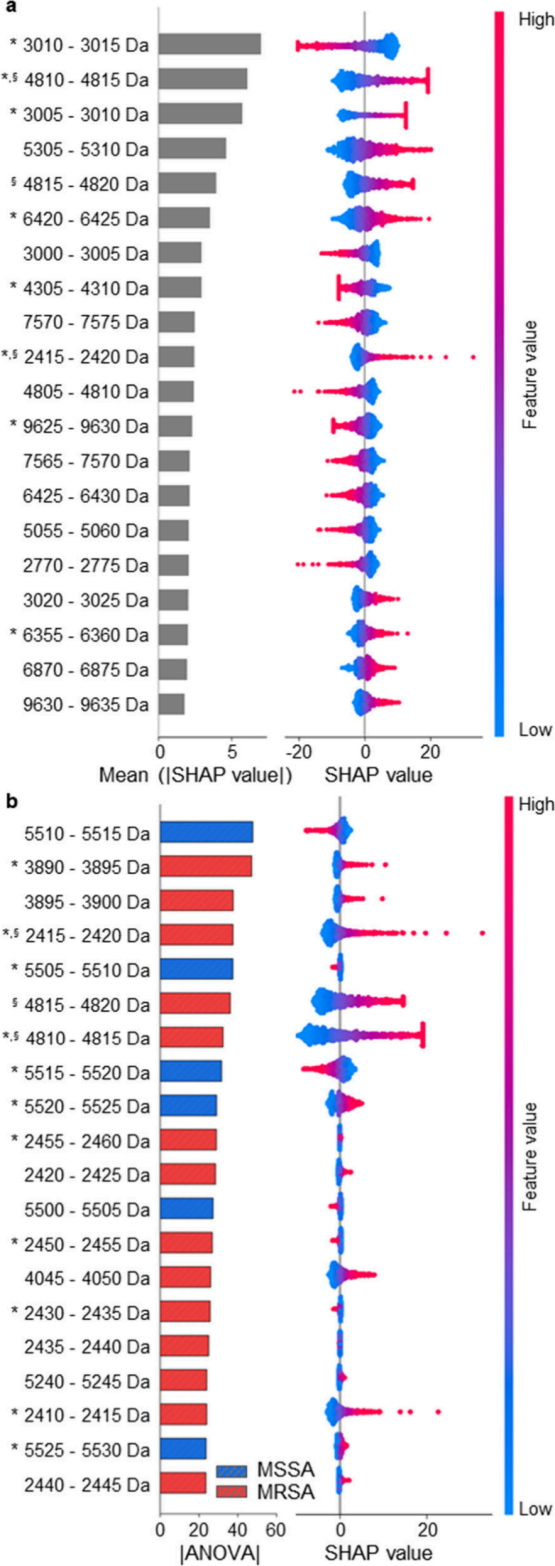
Contribution of feature *m*/*z* ranges to MRSA screening. (a) Shapley additive explanations (SHAP) values of the 20 most impactful feature *m*/*z* ranges. A positive SHAP value represents the contribution to the determination of MRSA. (b) ANOVA results of the top 20 features ordered by magnitude of upregulation in MRSA or MSSA and their SHAP plot. * Previously identified *m*/*z* features. ^§^ *m*/*z* features in the top 20 for both ANOVA and SHAP analysis.

Various proteins have been suggested as markers for distinguishing between MRSA and MSSA. The targets identified for the feature *m*/*z* ranges that showed a high contribution to SHAP and ANOVA analyses are listed in Table S2, Supporting Information. Among the 38 significant features (top 20 feature *m*/*z* ranges in SHAP and ANOVA), 17 features, including the phenol-soluble modulin (PSM)-*mec* peptide,^6,22,23^ formylated delta-toxin,^22,24–26^ 50S ribosomal proteins L30, L32, and L36,^25–27^ DNA-binding protein HU,^27,28^ uncharacterized proteins,^25,27^ and unidentified mass peaks indicating differences in MRSA and MSSA,^28–33^ were reported in previous studies. In particular, the uncharacterized protein SA2420.1 (*m*/*z* 3890–3895), PSM-*mec* peptide (*m*/*z* 2415–2420), and SAS049 (*m*/*z* 5505–5510) were ranked second, fourth, and fifth, respectively, in ANOVA analysis, whereas the DNA-binding protein HU (*m*/*z* 4810–4815) was ranked second only in SHAP analysis. The PSM-*mec* peptide (*m*/*z* 2415), a known signature biomarker of MRSA, ranked 10th in the SHAP analysis, and 13 of the top 20 features identified by SHAP analysis have not yet been identified. These results indicate the existence of additional biomarkers or the possibility of mass shifts by modification of existing biomarkers due to methicillin resistance, which are important for multifeature-based MRSA screening.

### MRSA Screening of Testing Set Using AMRQuest Software

AMRQuest software was installed on the MicroIDSys LT MALDI-TOF MS system to enable loading of mass spectra within the *m*/*z* range 2,000–20,000 which were used for microbial identification of *S. aureus*. The results of the AMRQuest test were obtained with scores ranging from 0.0 to 1.0. The AMRQuest scores represent the likelihood of MRSA detection using a machine learning model. The results of MRSA screening using the AMRQuest score were compared with those of the cefoxitin disk diffusion test as a standard MRSA screening test, as shown in Figure 3. A violin plot was prepared using the AMRQuest scores of the test set, which included 231 MRSA and 306 MSSA isolates. The AMRQuest scores of MRSA isolates had a higher median value (0.99995) than those of MSSA isolates (1.82498 × 10^–4^). The Mann–Whitney unpaired test confirmed that MRSA and MSSA were clearly categorized using the AMRQuest score (*P* < 0.0001), with only seven isolates being classified differently using the cefoxitin disk diffusion test. Three *S. aureus* isolates were found to be in the gray zone: one false negative, one true positive, and one true negative according to a cutoff score. Receiver operating characteristic (ROC) and precision-recall (PR) curves were obtained using the AMRQuest score, and the results of MRSA determination were obtained using reference methods. The *S. aureus* isolates with a score in the gray zone were considered false positives or false negatives. The areas under the ROC curve (AUROC) and PR curve (AUPRC) were estimated to be 0.979 and 0.973, respectively (Figure 3c, d). In addition, when the cutoff value for screening MRSA using AMRQuest was set to >0.5, the sensitivity, specificity, precision, and recall of AMRQuest for the testing set were 98.7%, 97.7%, 97.4%, and 98.7%, respectively. Using the cefoxitin disk diffusion test as a reference method, the PPVs and NPVs of the AMRQuest test using 537 *S. aureus* isolates were 97.4% and 99.7%, respectively. The percent positive agreement (PPA; sensitivity), percent negative agreement (PNA; specificity), and overall percent agreement of AMRQuest were 98.7%, 97.7%, and 98.1%, respectively. Cohen’s kappa coefficient, which evaluates the level of agreement between AMRQuest and the reference method, was 0.96. These results show that the AMRQuest test can be used for rapid MRSA screening with high clinical performance, consistent with the reference MRSA screening method.

**Figure 3:**
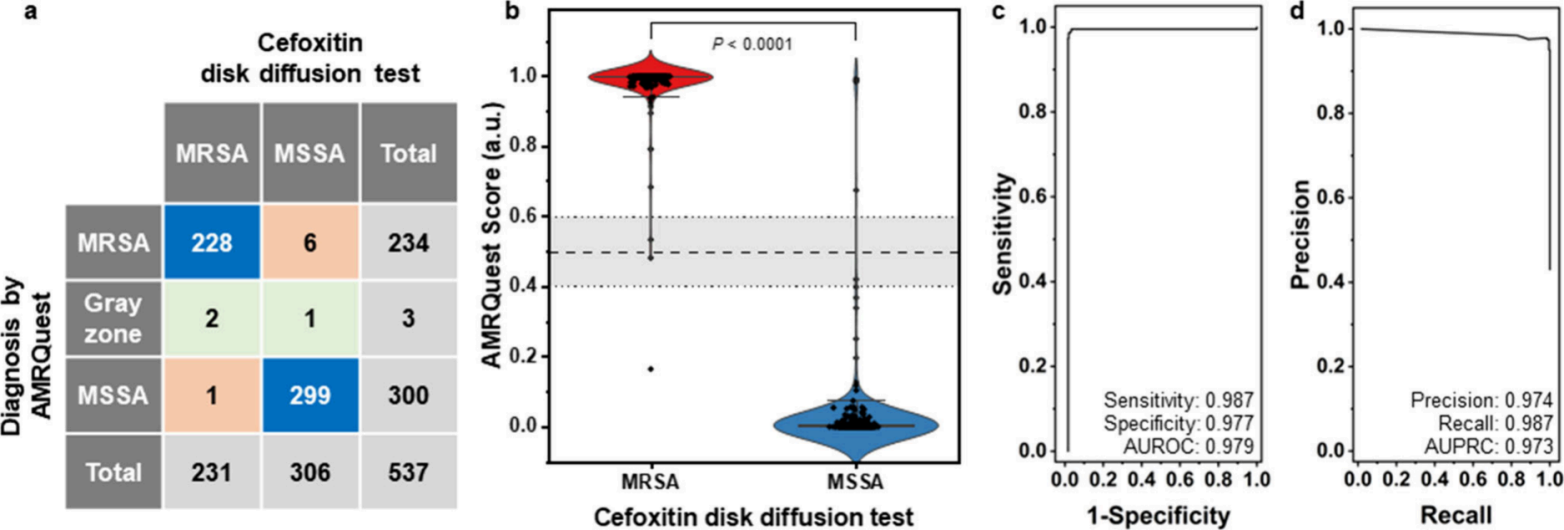
MRSA screening performance of AMRQuest software. (a) Confusion matrix, (b) Violin plot, (c) the receiver operating characteristic curve, and (d) the precision-recall curve of analysis results using AMRQuest software from testing set including MRSA (*n* = 231) and MSSA (*n* = 306). The whiskers in violin plot indicated the 5th and 95th percentiles. The horizontal dotted line (score = 0.5) represents the cutoff AMRQuest score. The score range of 0.4 to 0.6, indicated by the gray zone, represents the low-confidence prediction range. The ROC curve and the PR curve were plotted considering that the *S. aureus* isolates with the AMRQuest score in the gray zone were false positives or false negatives. AUROC, area under the receiver operating characteristic curve; AUPRC, area under the precision-recall curve.

In terms of screening, a high PPV is required to decrease the false-positive ratio, because a high false-positive ratio is one of the reasons for unnecessary overtreatment and the induction of antibiotic resistance. In a previous study based on a mathematical model simulation, the importance of PPV as a diagnostic criterion increased with a higher prevalence of the disease.^34^ The simulation showed that the PPV increased from 50% to 90% when the prevalence increased from 5% to 50%. The prevalence of MRSA infection in Korea from 2016 to 2017 was reported to be 43.25% according to the Korea Disease Control and Prevention Agency.^21^ The results of this study, including 97.4% PPV and 99.7% NPV, indicate that the AMRQuest software fulfills the PPV and NPV calculated from the meta-analysis. Moreover, the AMRQuest software showed high PPV and NPV regardless of where the bacteria were collected and evaluated. The PPV of the AMRQuest MRSA screening test using *S. aureus* isolates collected at Yonsei University Severance Hospital, Hallym University Kangdong Sacred Heart Hospital, and Seoul National University Bundang Hospital were 98.3%, 97.1%, and 97.1%, respectively, whereas the NPV were 100%, 97.1%, and 100%, respectively, indicating consistent clinical performance regardless of the collection and testing location (Table S3, Supporting Information). The AMRQuest software, which was embedded into the MicroIDSys LT MALDI-TOF MS system, loaded the mass spectrum of bacteria directly. Then, *S. aureus* identification and MRSA screening can then be performed simultaneously. In terms of turnaround time, conventional phenotypic antimicrobial susceptibility testing methods, including broth microdilution, disk diffusion testing, modified Hodge testing, and automated devices, require more than 1 day after bacterial identification. In the case of molecular diagnostics, it cannot be used when the resistance gene is not well characterized. However, in the case of SCC*mec* in *S. aureus*, antimicrobial susceptibility testing is possible within 2 to 4 h after bacterial identification and initial culture. In this study, methicillin-susceptibility testing using AMRQuest could be completed within a few seconds, immediately after MALDI-TOF MS bacterial identification. These results indicate that the AMRQuest software can be used for the rapid and accurate screening of MRSA infections before conventional antimicrobial susceptibility testing.

### SCC*mec*A PCR Gel Electrophoresis

To confirm the discrepancy between the cefoxitin disk diffusion test and AMRQuest MRSA screening, the *mec*A gene was detected using PCR gel electrophoresis. Among 231 MRSA isolates, 117, 4, 39, and 70 *mec*A genes of types II, III, IV, and IVA, respectively, were detected. The SCC*mec* type of cefoxitin-resistant *S. aureus* strain, which was identified as MSSA by AMRQuest, was confirmed to be MRSA with SCC*mec* type IV. In addition, six cefoxitin-susceptible *S. aureus* strains screened for MRSA using AMRQuest were confirmed to be MSSA. For *S. aureus* in the gray zone, the results of the cefoxitin disk diffusion test and the SCC*mec* test were consistent.

### Evaluation of Clinical Performance of AMRQuest Software

Recently, various studies on MRSA screening using MALDI-TOF MS based on machine learning models have been conducted (Table 1). To apply machine learning to mass spectrometry data, significant mass peaks can be selected to remove potential noise peaks that may induce a lower discriminating power. Wang et al. selected a feature peak set consisting of 193 mass peaks ranging from *m*/*z* 2000 to 20000, using the sequential forward selection method after evaluating all mass peaks using the Pearson correlation coefficient and one-rule strategy.^31^ Similarly, Liu et al. selected 38 mass peaks using least absolute shrinkage and selection operator regression.^35^ The selection of a small number of feature mass peaks for MRSA screening requires consistent updating and evaluation of the feature list to ensure that the emerging MRSA groups can be screened. In contrast, in several studies, including the present one, the mass spectrum ranging from *m*/*z* 2000 to 20000 was binned into fixed *m*/*z* intervals, and the sums^4^ or normalized averages^36^ of the bins were used as features. Kong et al., used a modified binning method by adjusting the bin size according to the peak width.^37^ Bin size can be optimized to improve the clinical performance of MRSA screening.^31^ However, reducing the bin size is not the only strategy because of the low resolution of MALDI-TOF MS, which can affect feature selection. Machine learning models, including decision trees, random forests, k-nearest neighbors, support vector machines, light gradient boosting machines, logistic regression, multilayer perceptron, and polynomial regression, have been evaluated in various studies^4,18,31,35–37^ and all showed AUCs above 0.75, up to 0.91. The logistic regression-based AMRQuest software used in this study showed significantly higher clinical performance than previous studies and improved results compared to the previous version based on the random forest model with small cohorts.^18^

**Table 1:** Previous studies on MRSA screening using MALDI-TOF MS based on machine learning^a^

Sample size^b^							
MRSA	MSSA	Feature size	Machine learning model	Sensitivity (%)	Specificity (%)	AUC	Reference
Selected Mass Peak Features							
732	788	193	DT	69.8	72.8	0.750	Wang et al. (2021)^31^
			RF	76.5	76.5	0.849	
			KNN	75.3	75	0.829	
			SVM	74.2	74.2	0.811	
194	258	38	RBF-SVM	84	88	0.89	Liu et al. (2021)^35^
			RF	74	88	0.87	
Features with Mass Spectra Binned into Fixed m/z Intervals							
72	110	508^c^	SVM	75.0	95.5	0.866	Kong et al. (2022)^37^
			DT	72.2	80	0.796	
			RF	68.1	81.8	0.824	
			PR	51.4	91.8	0.824	
NA^d^	NA	6,000 (bin size 3)	LightGBM	NA	NA	0.80	Weis et al. (2022)^4^
			LR	NA	NA	0.75	
			MLP	NA	NA	0.79	
8305^e^	6252	1,800 (bin size 10)	LightGBM	72–83^f^	65–88	0.78–0.91	Yu et al. (2022)^36^
106	88	3,600 (bin size 5)	RF	91.8	83.3	0.876	Jeon et al. (2022)^18^
231	306	3,600 (bin size 5)	LR	98.7	97.7	0.979	This study

a Features for machine learning were selected from mass peaks or set as the sum of intensities of fixed-interval bins of mass spectra in the *m/z* 2,000–20,000 range. Abbreviations: MRSA, methicillin-resistant *S. aureus*; MSSA, methicillin-susceptible *S. aureus*; AUC, area under the receiver characteristic curve; DT, decision tree; RF, random forest; KNN, K-nearest neighbor; SVM, support vector machine; LightGBM, light gradient boosting machine; LR, logistic regression; MLP, multilayer perceptron; RBF, radial basis function; PR, polynomial regression; NA, not available.

b Testing set only.

c Variable bin size according to the peak width.

d Data set over 300,000 mass spectra profiles and 750,000 antimicrobial resistance phenotypes from four medical institutions;

e Sum of isolates collected from five hospitals in China and Taiwan.

f Clinical performance from individual evaluations at five hospitals.

As shown in Figure 2, the feature *m*/*z* ranges for screening MRSA using the logistic regression model were mainly distributed from *m*/*z* 2410 to 9635. Among the feature *m*/*z* ranges, *m*/*z* 3000–3010 and 3895–3900 had the highest AUC of 0.641 (*P* < 0.0001), and the *m*/*z* 2,415–2,420 range, including the PSM-*mec* peptide in some MRSA cases, had an AUC of 0.599 (*P* = 0.0001), as shown in Table S4 (Supporting Information). Any individual feature, including the *m*/*z* ranges 3010–3015 and 5510–5515, which exhibited the highest contributions in the SHAP and ANOVA analyses, respectively, was found to have an insufficient AUC (0.614 and 0.631, respectively) for MRSA screening. Additionally, the sensitivity and specificity of each individual feature *m*/*z* range were calculated using Youden *J* statistics for each ROC curve.^38^ For example, the sensitivity and specificity of *m*/*z* 3010–3015 were 45.45% and 74.51%, respectively, whereas those of *m*/*z* 5510–5515 were 85.28% and 35.95%, respectively. As shown in Figures S1–2 (Supporting Information), although MRSA and MSSA could be distinguished by most feature *m*/*z* ranges with significant *p* values, when any certain cutoff intensity was set, significant false positives and false negatives were observed. These results indicated that neither feature *m*/*z* range possessed sufficient clinical performance for use as a single marker for MRSA screening. In contrast, AMRQuest software with a logistic regression model using multiple feature *m*/*z* ranges showed excellent clinical performance, with an AUC of 0.979, sensitivity of 98.7%, specificity of 97.7%, and positive and negative predictive values of 97.4% and 99.6%, respectively, for the 537 *S. aureus* isolates in the testing set. Moreover, to evaluate the agreement level between the cefoxitin disk diffusion test as the reference MRSA screening method and the AMRQuest software, Cohen’s kappa (*K*) was used as a statistical method to evaluate agreement between 0 and 1.^39^ *K* = 0 indicates completely different evaluations, and *K* = 1 indicates perfect agreement. In this study, Cohen’s kappa for AMRQuest was 0.96, indicating near-perfect agreement. Therefore, the AMRQuest, which is based on a logistic regression model, can be considered an effective method for MRSA screening.

Nevertheless, our study has some limitations that should be considered. There were insufficient cases of discrepancy identified from the SCC*mec* analysis to determine the cause of false positives and false negatives in the AMRQuest software. In all eight cases of discrepancy between the cefoxitin disk diffusion test and the results of the AMRQuest MRSA screening, the cefoxitin disk diffusion test was confirmed to be correct by SCC*mec* type analysis. These results indicate that the cefoxitin disk diffusion test can be used to correctly detect MRSA; however, further studies are required to determine the cause of this discrepancy. Although more *S. aureus* isolates were collected for AMRQuest than the minimum number to achieve statistical significance based on prevalence and meta-analysis, further evaluation is needed on *S. aureus* samples from various regions. The feasibility of AMRQuest to identify resistant to methicillin for bacterial species other than *S. aureus* has not yet been validated. In this study, MRSA screening was performed using MALDI-TOF mass spectra that were subsequently confirmed to be *S. aureus* after bacterial identification, because AMRQuest software was trained with only *S. aureus*. Consequently, it was essentially impossible for a mass spectrum from a non-*S. aureus* isolate to be loaded into the AMRQuest system. In the future, if the scope of AMRQuest is expanded by adding other bacterial species and other antimicrobial resistance testing algorithms, it is necessary to assign the appropriate screening algorithm based on the results of the bacterial identification.

## Conclusion

In summary, we presented the AMRQuest software based on MALDI-TOF MS and logistic regression as a rapid MRSA screening method. The results of MRSA screening by AMRQuest using 537 *S. aureus* isolates suggested that MRSA could be successfully identified with a high clinical predictive performance. Additionally, the AMRQuest results were similar to those of the cefoxitin disk diffusion test, which was used as the reference method. ANOVA and SHAP analyses were used to determine the contribution of each feature *m*/*z* range, and it was confirmed that multifeature-based screening with machine learning was more suitable for MRSA discrimination than single-marker analysis. Compared with previous studies that used various machine learning techniques, the AMRQuest software based on logistic regression showed significantly better performance. In conclusion, it is suggested that the AMRQuest software can be used as a rapid MRSA screening method in a clinical laboratory, and further studies are needed to determine the causes of the discrepancy from the reference method as well as to identify unknown features.

## Supplementary Material



## Data Availability

The source codes underlying this study are not publicly available due to proprietary constraints. The hyperparameters and feature extraction protocols are available from the corresponding author for reasonable noncommercial research purposes with institutional approval.

## Notes

Calculating the sample size for testing set; detailed procedure for MALDI-TOF mass spectrometry; Optimization and construction of AMRQuest; Figure S1: Box plot for feature *m*/*z* range selected by SHAP analysis; Figure S2: Box plot for feature *m*/*z* range selected by ANOVA analysis; Table S1: Comparison of various machine learning models for MRSA screening; Table S2: Ranked feature *m*/*z* ranges obtained from SHAP and ANOVA analysis; Table S3: Clinical performances of AMRQuest software according to the collection and testing location; Table S4: Clinical performances of AMRQuest software for each feature *m*/*z* range (PDF)
