# Supplementary material for: Rapid Screening of Methicillin-Resistant Staphylococcus aureus Using MALDI-TOF MS and Machine Learning: A Randomized, Multicenter Study
Source: Anal Chem. 2025 Jul 16;97(29):15667–75. doi: 10.1021/acs.analchem.5c01286 (PMC12311893; doi:10.1021/acs.analchem.5c01286)
Supplement: Supplementary file 1 [file ac5c01286_si_001.pdf]

Supporting Information

Rapid Screening of Methicillin-resistant *Staphylococcus aureus* using MALDI-TOF MS and Machine Learning: A Randomized, Multicenter Study

Dongeun Yong[a,‡], Jeong Su Park[b,‡], Kyungnam Kim[a], Donggun Seo[c], Dong-Chan Kim[c], Jae-Seok Kim[d,*], Jong-Min Park[e,f,*]

[a] Department of Laboratory Medicine and Research Institute of Bacterial Resistance, Yonsei University College of Medicine, Seoul 03722, Republic of Korea

[b] Department of Laboratory Medicine, Seoul National University Bundang Hospital, Seoul National University College of Medicine, Gyeonggi-do 13620, Republic of Korea

[c] NQ-LAB Co., ltd., Gyeonggi-do 16827, Republic of Korea

[d] Department of Laboratory Medicine, Kangdong Sacred Heart Hospital, Hallym University College of Medicine, Seoul 05355, Republic of Korea

[e] Major in Materials Science and Engineering, School of Future Convergence, Hallym University, Gangwon-do 24252, Republic of Korea

[f] Interdisciplinary Program of Nano-Medical Device Engineering and Integrative Materials Research Institute, Hallym University, Gangwon-do 24252, Republic of Korea

*Corresponding authors: Jae-Seok Kim (jaeseokcp@gmail.com) and Jong-Min Park (jongminpark@hallym.ac.kr)

Table of Contents

1. Experimental details ········ S2
   A. Calculating the sample size for testing set ········ S2
   B. MALDI-TOF mass spectrometry ········ S3
   C. Optimization of machine learning model and construction of AMRQuest software ········ S3
2. Supplementary Figures ········ S5
3. Supplementary Tables ········ S7
4. References ········ S11

1. Experimental Details

A. Calculating the sample size for testing set

Clinical performance characteristics, including sensitivity, specificity, PPV, and NPV, were used to determine the optimal sample size for the clinical evaluation of MRSA screening using the AMRQuest software. These values were calculated using a meta-analysis of MRSA screening results from 22 different studies and 5,163 patients with MRSA pneumonia.[1] According to the meta-analysis results, the assumption of a 10% prevalence of potential MRSA pneumonia yielded a PPV of 44.8% and NPV of 96.5%, with pooled sensitivity and specificity values of 70.9% and 90.3%, respectively. In this study, the disease prevalence was 43.25% because all *S. aureus* isolates were obtained from blood cultures in Korean hospitals from 2016 to 2017.[2]

The total size of the sample n for the clinical study was calculated using PPV and NPV, disease prevalence in Korea, and the following equations: [3]

$$n = (z_{\alpha/2} + z_{\beta})^2 \frac{p(1-p)}{(p-p_0)^2} \qquad (1)$$

In the equation (1), z is the z-score in the standard normal distribution at the significance level α and the statistical power (1 – β), and p and p_0 represent the expected PPV or NPV and the minimum acceptable PPV or NPV, respectively. The expected PPV and NPV were calculated using the disease prevalence (43.3%), pooled sensitivity (70.9%), and specificity (90.3%) from a meta-analysis.[4] To estimate the minimum size of the negative (MSSA) sample, a null hypothesis was established, which states that the NPV of the AMRQuest test (p) is less than that of the meta-analysis of MRSA screening (p_0). The dropout rate was set to 5%. For the negative sample, the expected p value and lower bound of the 95% confidence interval p_0 values were 80.3% and 73.7%, respectively. The minimum negative sample size required to achieve the target NPV and to reject the null hypothesis was 299. Similarly, to estimate the size of the positive (MRSA) sample, another null hypothesis was established, which states that the PPV of the AMRQuest test (p) is less than the PPV from the meta-analysis of MRSA screening (p_0), and the dropout

rate is 5%. For the positive sample, the target *p* value and lower bound of the 95% confidence interval p_0 values were 84.8% and 70.3%, respectively. The number of positive samples required to achieve the desired PPV and reject the null hypothesis was calculated to be 51. Finally, the minimum size of the positive sample was adjusted to 226, considering the prevalence of MRSA infection in Korea, the ratio of MRSA to MSSA, and the calculated minimum size of the negative sample. According to the calculated sample size, 537 *S. aureus* strains

B. MALDI-TOF mass spectrometry

Mass spectra of *S. aureus* isolate were obtained using MicroIDSys® LT MALDI-TOF mass spectrometry system (ASTA, Suwon, Korea) equipped with a nitrogen laser (337 nm), according to the manufacturer's instructions. A single colony from each strain was smeared onto an ID plate (ASTA, Suwon, Korea), followed by the application of 1.5 μL of 70% formic acid and subsequent drying. After adding 1.5 μL of α-cyano-4-hydroxycinnamic acid (CHCA; ASTA, Suwon, Korea) matrix and drying, MALDI-TOF mass spectra were generated in linear positive ion mode from *m/z* (mass-to-charge ratio) 2,000 to 20,000, with a first accelerating voltage of 18.0 kV and a second ion source voltage of 16.8 kV. All mass spectra were obtained by integrating 1,200 laser pulses for each sample spot with random motion. The mass spectra were analyzed using MicroID CoreDB version 1.27.04 and calibrated with AMS Calibration Standard Material (ASTA, Suwon, Korea). *S. aureus* isolates were identified with the cut-off score setting at ≥ 140 according to the manufacturer's recommendation.

C. Optimization of machine learning model and construction of AMRQuest software

The AMRQuest software was developed to integrate into clinical workflows and operate independently. Similarly to MALDI-TOF MS-based bacterial identification systems that are widely used in clinical microbiology laboratories, AMRQuest contains a comprehensive set of components, including data

preprocessing to extract MALDI-TOF mass features, model prediction to generate AMRQuest scores based on logistic regression, graphic user interfaces and integrative functions compatible with laboratory information systems (LIS). To optimize AMRQuest software, the MRSA screening performance of six machine learning model algorithms, including logistic regression, neural networks, gradient boosting, random forest, decision tree, and support vector machine, was evaluated using the AUC, CA, F1 score, precision, and recall values. The significance level α and the statistical power (1 – β) was set to 0.05 and 0.8, respectively, for all tests. As shown in Supplementary Table S1, among the six machine learning models, the highest performance, including an area under the receiver operating characteristic curve (AUC), classification accuracy (CA), F1 score, precision, and recall of 0.999, 0.986, 0.986, 0.986, and 0.986, respectively, was obtained using logistic regression. Therefore, a logistic regression algorithm was adopted in the AMRQuest software after optimization using specific MALDI-TOF MS hyperparameters.

The AMRQuest software was entirely constructed using Python 3.8-based tools and libraries, including Django, DRF (Django REST Framework), Django-filter, Djoser, pyinstaller, drf-yasg, Django-cors-headers, PostgreSQL, and logic. Data analysis and logistic regression machine learning models were configured using NumPy, Pandas, and scikit-learn libraries. The graphical user interface was developed using the PyQt framework.

2. Supplementary Figures

Supplementary Figure S1. Box plot of MALDI-TOF mass intensities for each feature *m/z* range that was listed in order of the highest contribution in the SHAP analysis.

(*, p<0.05; **, p<0.01; ***, p<0.001; ns, not significant)

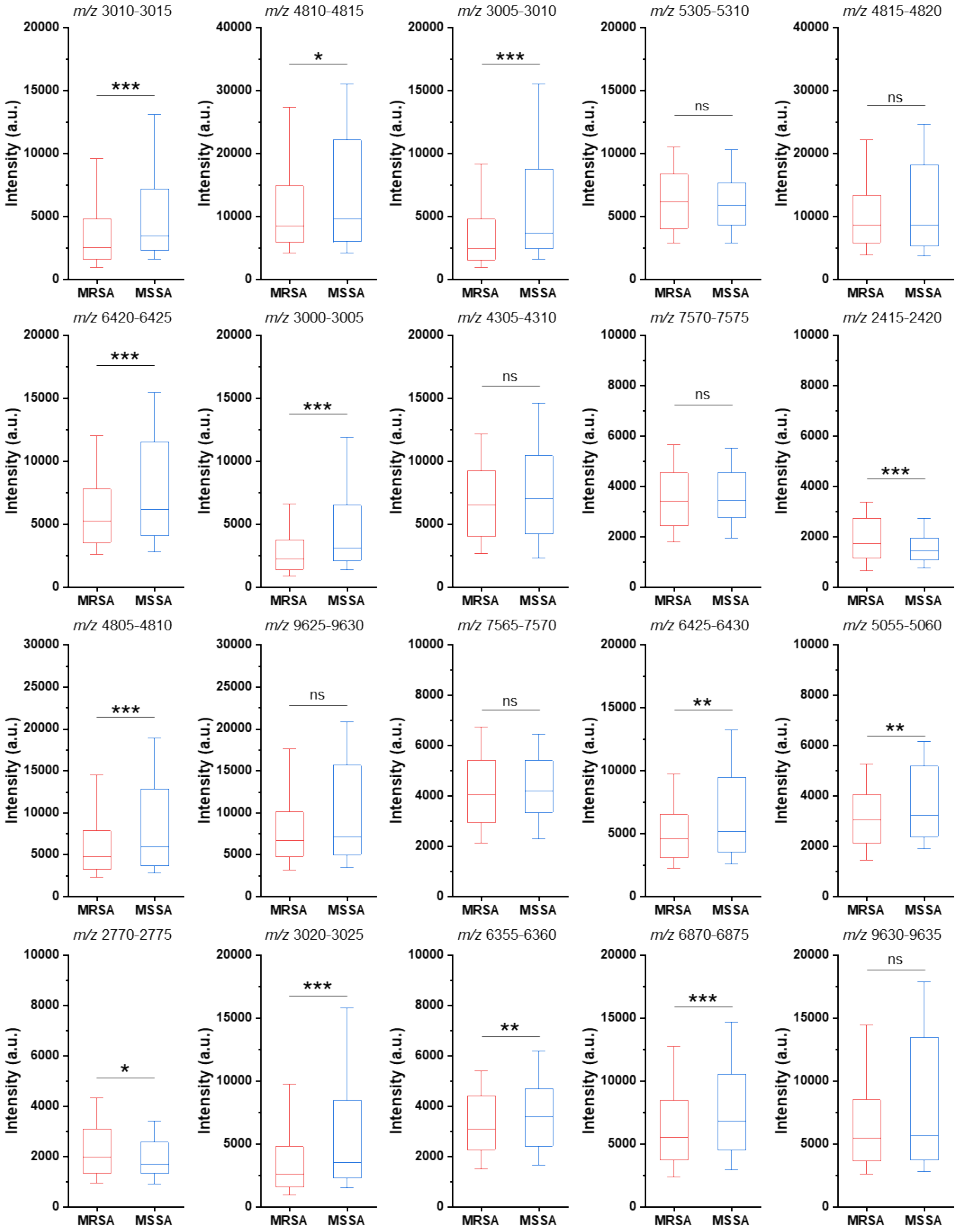

Supplementary Figure S2. Box plot of MALDI-TOF mass intensities for each feature *m/z* range that was listed in order of the highest contribution in the ANOVA analysis. (*, $p<0.05$; **, $p<0.01$; ***, $p<0.001$; ns, not significant)

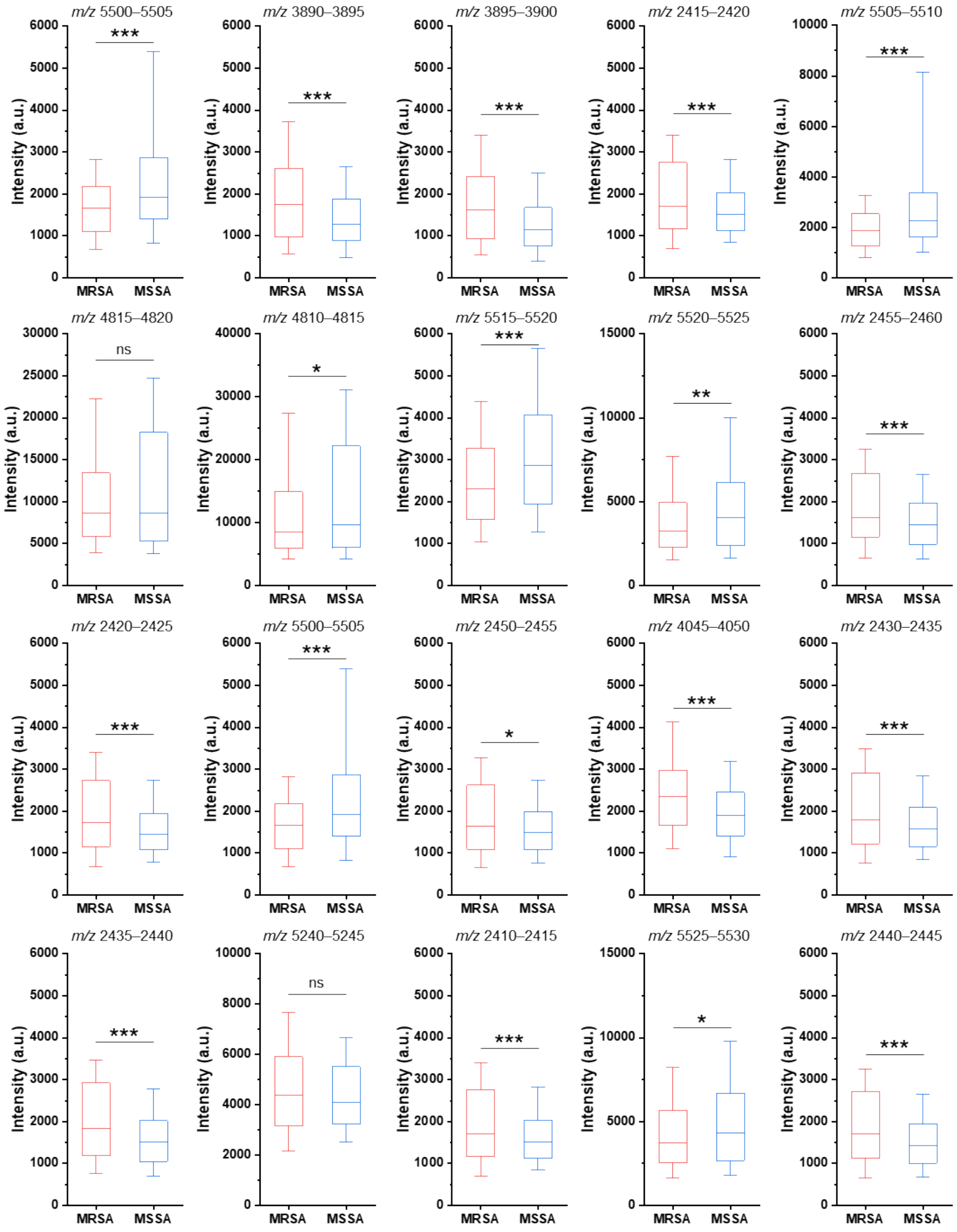

3. Supplementary Tables

Supplementary Table S1. Comparison of various machine learning models for MRSA screening using training set.

Model	AUC	CA	F1 score	Precision	Recall
Logistic regression	0.999	0.986	0.986	0.986	0.986
Neural network	0.990	0.960	0.960	0.960	0.960
Gradient boosting	0.961	0.905	0.905	0.905	0.905
Random forest	0.890	0.806	0.806	0.808	0.806
Decision tree	0.813	0.803	0.803	0.804	0.803
Support vector machine	0.859	0.734	0.725	0.759	0.734

Abbreviations: AUC, area under the receiver operating characteristic curve; CA, Classification Accuracy.

Supplementary Table S2. Ranked feature *m/z* ranges obtained from SHAP and ANOVA analysis in this study. Exact *m/z* values and the targets that have been identified were obtained in previous studies. For references where the *m/z* was not clearly specified, the exact *m/z* was labeled as a range. The feature m/z ranges that were not referred to represent those that were the first found in this study.

Feature *m/z* range	SHAP rank	ANOVA rank	Target	Exact *m/z*
2410–2415		18	Not identified	2410–2415[5,6]
2415–2420	10	4	PSM-mec peptide	2415[7,8]
2420–2425		11		
2430–2435		15	Not identified	2430–2440[9]
2435–2440		16	Not identified	2430–2440[9]
2440–2445		20		
2450–2455		13	Related to the presence of the *mec*-element	2450[10], 2450–2455[5,6]
2455–2460		10	Not identified	2455–2460[6]
2770–2775	16			
3000–3005	7			
3005–3010	3		Delta-toxin (formylated)	3007[7,11-13], 3008[14]
3010–3015	1			
3020–3025	17			
3890–3895		2	Uncharacterized protein SA2420.1	3891[12], 3890[14]
			Typical peak shift for CC5 strains	3891[7,11-13]
3895–3900		3		
4045–4050		14		
4305–4310	8		50S ribosomal protein L36	4306[12,15]
4805–4810	11			
4810–4815	2	7	DNA-binding protein HU	4814[14,15]
4815–4820	5	6		
5055–5060	15			
5240–5245		17		
5305–5310	4			
5500–5505		12		
5505–5510		5	Uncharacterized protein ORF SAS049	5507[12], 5506[14]
			Marker for spa type t021	5507[13,16]
5510–5515		1		
5515–5520		8	Marker for spa type t019	5516[13]
5520–5525		9	Not identified	5523[14], 5524[17]
5525–5530		19	Uncharacterized protein ORF SAS049	5525[12,15]
6355–6360	18		50S ribosomal protein L32	6354[12,15]
6420–6425	6		50S ribosomal protein L30	6423[12,13]
6425–6430	14			
6870–6875	19			
7565–7570	13			
7570–7575	9			
9625–9630	12		DNA-binding protein HU	9627[15]
9630–9635	20			

Supplementary Table S3. Clinical performances of AMRQuest software according to the collection and testing location. *S. aureus* isolates that obtained the AMRQuest score between 0.4 and 0.6 were classified as gray zone. The gray zone values were treated as false negatives and false positives in the clinical performance calculations.

Diagnosis by AMRQuest	Cefoxitin Disk Diffusion Test			PPV	NPV	PPA (Sensitivity)	PNA (Specificity)	Cohen's Kappa
	MRSA	MSSA	Total					
Seoul National University Bundang Hospital								
MRSA	135	4	139	97.1%	100.0%	100.0%	97.9%	0.97
Gray zone	0	1	1					
MSSA	0	230	229					
Total	135	234	369					
Yonsei University Severance Hospital								
MRSA	59	1	60	98.3%	100.0%	96.7%	97.3%	0.94
Gray zone	2	0	2					
MSSA	0	36	36					
Total	61	37	98					
Hallym University Kangdong Sacred Heart Hospital								
MRSA	34	1	35	97.1%	97.1%	97.1%	97.1%	0.94
Gray zone	0	0	0					
MSSA	1	34	35					
Total	35	35	70					

Supplementary Table S4. Clinical performances of AMRQuest software for each feature *m/z* range that was listed in order of highest AUC.

SHAP rank	ANOVA rank	Feature *m/z* range	AUC	*P* value	Sensitivity (%)	Specificity (%)
7		3000 - 3005	0.641	<0.0001	40.26	83.66
3		3005 - 3010	0.641	<0.0001	48.92	76.47
	3	3895 - 3900	0.641	<0.0001	58.87	68.3
17		3020 - 3025	0.632	<0.0001	46.75	75.82
	1	5510 - 5515	0.631	<0.0001	85.28	35.95
	5	5505 - 5510	0.628	<0.0001	81.82	38.89
	2	3890 - 3895	0.619	<0.0001	59.31	64.05
	14	4045 - 4050	0.619	<0.0001	40.69	82.03
1		3010 - 3015	0.614	<0.0001	45.45	74.51
	8	5515 - 5520	0.614	<0.0001	56.71	63.07
	16	2435 - 2440	0.611	<0.0001	39.83	82.03
	12	5500 - 5505	0.611	<0.0001	79.22	38.24
10	4	2415 - 2420	0.599	0.0001	48.05	71.90
	20	2440 - 2445	0.599	0.0001	40.26	80.72
	10	2455 - 2460	0.594	0.0002	34.20	84.64
11		4805 - 4810	0.586	0.0005	77.49	40.20
	15	2430 - 2435	0.585	0.0009	38.53	81.05
19		6870 - 6875	0.584	0.0007	65.80	50.00
	18	2410 - 2415	0.583	0.0012	37.66	80.72
6		6420 - 6425	0.583	0.0007	83.98	33.33
	9	5520 - 5525	0.58	0.0012	75.76	40.85
	11	2420 - 2425	0.579	0.0022	36.80	82.68
15		5055 - 5060	0.579	0.0015	86.58	29.08
14		6425 - 6430	0.577	0.0017	80.95	36.27
18		6355 - 6360	0.569	0.0056	56.28	58.50
	13	2450 - 2455	0.564	0.0127	38.96	78.43
	19	5525 - 5530	0.564	0.0105	67.53	45.10
16		2770 - 2775	0.555	0.0317	48.05	63.73
2	7	4810 - 4815	0.55	0.0456	78.79	37.25
12		9625 - 9630	0.548	0.0548	76.19	39.22
20		9630 - 9635	0.543	0.085	79.22	37.58
8		4305 - 4310	0.541	0.0957	94.37	16.01
13		7565 - 7570	0.527	0.2908	37.23	75.49
	17	5240 - 5245	0.526	0.309	14.72	94.77
9		7570 - 7575	0.525	0.3254	35.06	76.80
5	6	4815 - 4820	0.515	0.539	81.82	33.33
4		5305 - 5310	0.515	0.5648	35.50	74.18

4. References

(1) Parente D. M.; Cunha C. B.; Mylonakis E.; Timbrook T. T. The clinical utility of methicillin-resistant *Staphylococcus aureus* (MRSA) nasal screening to rule out MRSA pneumonia: a diagnostic meta-analysis with antimicrobial stewardship implications. Clin. Infect. Dis. 2018, 67, 1–7.

(2) Lee S.; Lee E.; Bahk H.; Lee S.; Kim S.; Lee H. *Public Health Wkly. Rep.* **2019**, *12*, 485-490. https://www.phwr.org/journal/archives_view.html?eid=Y29udGVudF9udW09OTEy

(3) Banoo S.; Bell D.; Bossuyt P.; Herring A.; Mabey D.; Poole F.; Smith P. G.; Sriram N.; Wongsrichanalai C.; Linke R.; O'Brien R.; Perkins M.; Cunningham J.; Matsoso P.; Nathanson C. M.; Olliaro P.; Peeling R. W.; Ramsay A. Evaluation of diagnostic tests for infectious diseases: general principles. *Nat. Rev. Microbiol.* **2006**, *4*, S20–S32.

(4) Altman D. G.; Bland J. M. *BMJ* **1994**, *309*, 102.

(5) Bernardo K.; Pakulat N.; Macht M.; Krut O.; Seifert H.; Fleer S.; Hünger F.; Krönke M. Identification and discrimination of *Staphylococcus aureus* strains using matrix-assisted laser desorption/ionization-time of flight mass spectrometry. *Proteomics* **2002**, *2*, 747–753.

(6) Du Z.; Yang R.; Guo Z.; Song Y.; Wang J. Identification of *Staphylococcus aureus* and determination of its methicillin resistance by matrix-assisted laser desorption/ionization time-of-flight mass spectrometry. Anal. Chem. 2002, 74, 5487–5491.

(7) Josten M.; Dischinger J.; Szekat C.; Reif M.; Al-Sabti N.; Sahl H.-G.; Parcina M.; Bekeredjian-Ding I.; Bierbaum G. Identification of *agr*-positive methicillin-resistant *Staphylococcus aureus* harbouring the class A *mec* complex by MALDI-TOF mass spectrometry. *Int. J. Med. Microbiol.* **2014**, *304*, 1018–1023.

(8) Van Belkum A., Welker M., Pincus D., Charrier J.-P., Girard V. Matrix-assisted laser desorption ionization time-of-flight mass spectrometry in clinical microbiology: what are the current issues? *Ann. Lab. Med.* **2017**, *37*, 475–483.

(9) Wang H.-Y.; Chung C.-R.; Wang Z.; Li S.; Chu B.-Y.; Horng J.-T.; Lu J.-J.; Lee T.-Y. A large-scale investigation and identification of methicillin-resistant *Staphylococcus aureus* based on peaks binning of matrix-assisted laser desorption ionization-time of flight MS spectra. *Brief. Bioinform.* **2021**, *22*, bbaa138.

(10) Majcherczyk P. A.; McKenna T.; Moreillon P.; Vaudaux P. The discriminatory power of MALDI-TOF mass spectrometry to differentiate between isogenic teicoplanin-susceptible and teicoplanin-resistant strains of methicillin-resistant *Staphylococcus aureus*. *FEMS Microbiol. Lett.* **2006**, *255*, 233–239.

(11) Hu Y.; Huang Y.; Lizou Y.; Li J.; Zhang R. Evaluation of *Staphylococcus aureus* subtyping module for methicillin-resistant *Staphylococcus aureus* detection based on matrix-assisted laser desorption ionization time-of-flight mass spectrometry. *Front. Microbiol.* **2019**, *10*, 2504.

(12) Josten M.; Reif M.; Szekat C.; Al-Sabti N.; Roemer T.; Sparbier K.; Kostrzewa M.; Rohde H.; Sahl H.-G.; Bierbaum G. Analysis of the matrix-assisted laser desorption ionization–time of flight mass spectrum of *Staphylococcus aureus* identifies mutations that allow differentiation of the main clonal lineages. *J. Clin. Microbiol.* **2013**, *51*, 1809-1817.

(13) Østergaard C.; Hansen S. G.; Møller J. K. Rapid first-line discrimination of methicillin resistant *Staphylococcus aureus* strains using MALDI-TOF MS. *Int. J. Med. Microbiol.* **2015**, *305*, 838–847.

(14) Böhme K.; Morandi S.; Cremonesi P.; Fernandez No I. C.; Barros-Velázquez J.; Castiglioni B.; Brasca M.; Cañas B.; Calo-Mata P. *Electrophoresis* **2012**, *33*, 2355-2364.

(15) Dekio I.; Sugiura Y.; Hamada-Tsutsumi S.; Murakami Y.; Tamura H.; Suematsu M. What Do we see in spectra? Assignment of high-intensity peaks of *Cutibacterium* and *Staphylococcus* spectra of maldi-tof mass spectrometry by interspecies comparative proteogenomics. *Microorganisms* **2021**, *9*, 1243.

(16) Sauget M.; van Der Mee-Marquet N.; Bertrand X.; Hocquet D. Matrix-assisted laser desorption ionization-time of flight Mass spectrometry can detect *Staphylococcus aureus* clonal complex 398. *J. Microbiol. Methods* **2016**, *127*, 20–23.

(17) Wolters M.; Rohde H.; Maier T.; Belmar-Campos C.; Franke G.; Scherpe S.; Aepfelbacher M.; Christner M. MALDI-TOF MS fingerprinting allows for discrimination of major methicillin-resistant *Staphylococcus aureus* lineages. *Int. J. Med. Microbiol.* **2011**, *301*, 64–68.
